# Prior Experience With Unlabeled Actions Promotes 3-Year-Old Children’s Verb Learning

**DOI:** 10.1037/xge0001071

**Published:** 2021-07-15

**Authors:** Suzanne Aussems, Katherine H. Mumford, Sotaro Kita

**Affiliations:** 1 Department of Psychology, University of Warwick; 2 BC Family Hearing Resource Society, Victoria, British Columbia, Canada

**Keywords:** verb learning and generalization, multiple exemplars, prior experience with unlabeled actions, iconic gestures, interactive gestures

## Abstract

This study investigated what type of prior experience with unlabeled actions promotes 3-year-old children’s verb learning. We designed a novel verb learning task in which we manipulated prior experience with unlabeled actions and the gesture type children saw with this prior experience. Experiment 1 showed that children (*N* = 96) successfully generalized more novel verbs when they had prior experience with unlabeled exemplars of the referent actions (“relevant exemplars”), but only if the referent actions were highlighted with iconic gestures during prior experience. Experiment 2 showed that children (*N* = 48) successfully generalized more novel verbs when they had prior experience with one relevant exemplar and an iconic gesture than with two relevant exemplars (i.e., the same referent action performed by different actors) shown simultaneously. However, children also successfully generalized verbs above chance in the two-relevant-exemplars condition (without the help of iconic gesture). Overall, these findings suggest that prior experience with unlabeled actions is an important first step in children’s verb learning process, provided that children get a cue for focusing on the relevant information (i.e., actions) during prior experience so that they can create stable memory representations of the actions. Such stable action memory representations promote verb learning because they make the actions stand out when children later encounter labeled exemplars of the same actions. Adults can provide top-down cues (e.g., iconic gestures) and bottom-up cues (e.g., simultaneous exemplars) to focus children’s attention on actions; however, iconic gesture is more beneficial for successful verb learning than simultaneous exemplars.

Figuring out the meaning of a novel verb is a challenging task for young children. Quine (1960) notes that even in ostensive situations, a novel verb could refer to an infinite number of referents. Children can use a range of both contextual cues (for temporal cues see Tomasello & Akhtar, 1995) and syntactic cues (for syntactic biases see Naigles & Kako, 1993, and for multiple exemplar learning see Maguire et al., 2008) to solve Quine’s (1960) problem for verb meaning. However, this referential ambiguity may also be reduced if children have encountered the verb’s referent before, even without hearing its label. Therefore, this study investigates whether prior experience with unlabeled actions promotes verb learning in 3-year-old children, and if so, what type of prior experience works best.

## Identifying Verb Referents Is a Challenging Task

Verbs typically describe actions, and it is difficult for children to individuate actions in complex events (Gentner, 1982). For example, 3-year-old children struggle to generalize verbs to events that show the referent actions performed by novel actors (e.g., Imai et al., 2008; Kersten & Smith, 2002), with novel objects (e.g., Imai et al., 2005), or with novel instruments (e.g., Behrend, 1990; Forbes & Farrar, 1993, 1995). This indicates that children’s semantic representations of verbs include components of action events that are irrelevant to verb meaning (i.e., actors, objects, instruments). In other words, children map verbs to the *combination* of event components, for example, to a particular actor performing a particular action or a particular action carried out with a particular object (e.g., Imai et al., 2005). Thus, if we can help children to individuate action components in complex events, then this could help children to learn verbs with semantic representations that include only the relevant component for verb meaning (Gentner, 2003). However, to achieve this, children need to segment complex events into the different event components (e.g., actors, objects, instruments, and crucially, actions). This study investigates three ways in which this could be achieved for novel intransitive verbs that describe manners of locomotion (actions) performed by adults (actors).

## Multiple Exemplars of the Same Action May Facilitate Verb Learning

The first way to help children hone in on actions in complex events is to present them with multiple labeled exemplars that consistently show the components that are relevant to verb meaning, while varying the components that are irrelevant to verb meaning (Childers, 2011; Haryu et al., 2011). For example, Childers (2011) taught 2.5-year-olds novel verbs while seeing the experimenter perform target action events (e.g., rolling a ball down a ramp into an opaque box so that the ball disappears from view) followed by either the repetition of these same labeled exemplar, labeled exemplars that repeated only the actions (e.g., rolling a ball down a curved tube), or labeled exemplars that repeated only the results (e.g., covering the ball with a piece of cloth so that it disappears from view). In the test phase, children were asked to enact the novel verb meanings with a set of objects that included the objects used in the target action events (e.g., a ramp), novel objects that could be used to enact the actions (e.g., a curved pipe), and novel objects that could be used to enact the results (e.g., an opaque bag). Children who saw similar labeled exemplars that repeated the actions were more likely to generalize the verbs to novel objects with which the same actions could be performed, and children who saw similar labeled exemplars that repeated the results were more likely to generalize the verbs to novel objects which led to the same results. In contrast, children who saw the repetition of the same labeled exemplar were conservative in generalizing the verbs as they were more likely to recreate the same event using the same objects. Thus, this study shows that when children are presented *sequentially* with multiple different labeled action exemplars, they can compare those exemplars and extract the consistent component that is shared between those exemplars, which is important for learning the meaning of that verb (i.e., manners, results). This ability to compare exemplars and extract relevant information facilitates children’s verb learning and generalization.

Previous research has shown that children can also learn verbs by integrating information from two different exemplars shown *simultaneously* (Snape & Krott, 2018). Snape and Krott (2018) taught 3-year-old children novel verbs, while some children saw a single exemplar of an action performed on a novel object, some saw two exemplars simultaneously in which the same action was performed on two different objects, and some saw two identical exemplars simultaneously in which no aspect of the events varied. In all three conditions, each exemplar was always labeled. In a two-alternative forced-choice test, the children were asked to extend the newly learned verbs to one of two events: one that maintained the action but performed on a novel object versus one that maintained the object but performed a novel action. Only children who saw two different labeled exemplars of the same action simultaneously (i.e., when the same action was performed on two different objects) successfully generalized the newly learned verbs to novel events that maintained the actions. This suggests that simultaneously presented exemplars can support 3-year-old children’s verb learning, but only when the content of the exemplars varies (i.e., the action component that is relevant for verb meaning is kept consistent across exemplars, but components irrelevant to verb meaning vary).

## Iconic Gestures That Encode Actions May Facilitate Verb Learning

The second way to help children to focus on actions in complex events is to highlight verb referents with iconic gestures (e.g., Goodrich & Hudson Kam, 2009; Mumford & Kita, 2014; Wakefield et al., 2018). Iconic gestures are hand movements that depict features of objects (e.g., shape) or actions (e.g., motion; McNeill, 1985, 1992). The shape and motion of an iconic gesture and its meaning are linked through similarity (e.g., wiggling the index and middle fingers to depict a person walking). As such, iconic gestures can focus children’s attention on components of complex events that are important for verb meaning. For example, Mumford and Kita (2014) taught 3-year-old children novel verbs that could be interpreted as manner verbs (e.g., “to push”) or result verbs (e.g., “to break”). Children saw videos of an actor manipulating material/objects (e.g., *sprinkling* sand into a *square* shape on a table surface) with either iconic gestures that highlighted manner (e.g., depicting the manual action of sprinkling) or iconic gestures that highlighted the end-state of the scene (e.g., tracing the square shape that the sand formed) while the experimenter labeled each action event with a novel verb. Children were immediately asked to generalize each novel verb to one of two novel scenes in a two-alternative forced-choice task: one scene showed a manner verb interpretation (e.g., *sprinkling* powder into a *triangle* shape) and the other a result verb interpretation (e.g., *placing* pieces of paper to form a *square*). Children who saw iconic gestures highlighting manner when the verbs were taught interpreted the verbs as manner verbs and children who saw iconic gestures highlighting end-state as result verbs. This suggests that iconic gestures can focus children’s attention on different components of complex events, and this influences children’s interpretation of novel verb meanings.

While there is abundant empirical evidence for the beneficial effect of iconic gesture on children’s word learning (Goodrich & Hudson Kam, 2009; McGregor et al., 2009; Mumford & Kita, 2014; Wakefield et al., 2018), the mechanism for how iconic gesture facilitates word learning is unclear. One possibility is that iconic gestures that depict actions merely function as an extra action exemplar, much like the two simultaneous exemplars in the study by Snape and Krott (2018). Another possibility is that iconic gesture goes beyond merely functioning as an extra exemplar because it *schematizes* action (Aussems & Kita, 2019, 2021; de Ruiter, 2000; Goldin-Meadow, 2015; Kita, 2000; Kita et al., 2017; Novack et al., 2014; Novack & Goldin-Meadow, 2017); that is, it provides children with a focused action representation. Iconic gesture is also a communicative signal, which prompts the recipient to search for a matching representation, triggering a top-down search for action.

## Prior Experience With Unlabeled Actions

The third way to help children focus on actions in complex events during verb learning is to give children prior experience with *unlabeled* referent actions. This is the main hypothesis of the current study. The word learning studies discussed so far leave it open whether *prior experience with unlabeled actions* can promote verb learning. In previous multiple exemplar studies, actions were always labeled on each encounter (e.g., Childers, 2011; Haryu et al., 2011; Imai et al., 2005; Maguire et al., 2008; Mumford & Kita, 2014); therefore, it is not clear whether children integrate prior experience with unlabeled actions in their semantic representations of novel verbs when they first encounter these action labels.

In a naturalistic verb learning situation, it is plausible that children encounter a referent action (along with many other actions) before they hear the label for that action for the first time. For example, children may have encountered actions like *galloping*, *slithering*, *leaping*, and *shrugging* before hearing a label for these actions. This is because adults frequently describe specific action events to young children using general all-purpose verbs (e.g., *to do*, *to go*), which are not tied to the specific actions in the events (Pinker, 1989; Rice & Bode, 1993). For example, in the nursery rhyme “Itsy Bitsy Spider,” the spider *went up* the waterspout. Although the verb *to crawl* is a commonly used verb to refer to a spider’s movement, it does not appear in the nursery rhyme, though many adults would probably intuitively depict the spider’s crawling motion in gesture (i.e., using the hand to represent the spider’s body and the fingers to represent its long legs). Moreover, the age of acquisition of the verb *to crawl* seems to be quite late, close to 4 years of age (Kuperman et al., 2012). This leaves room for children to gain experience with actions before hearing their specific verb labels, especially when iconic gesture is used to focus children’s attention on those actions. Thus, it is important to investigate whether and how children can take advantage of prior experience with unlabeled actions when they learn the labels for the actions at a later point in time. In the current study, we will emulate this understudied step of the verb learning process experimentally for the first time. In doing so, our study addresses a fundamentally different question than verb learning studies in which children were exposed to multiple labeled action exemplars (e.g., Childers, 2011; Haryu et al., 2011; Maguire et al., 2008). Such studies examined how *linguistic* representations of verbs change through encounters with multiple labeled exemplars of a referent action. However, our study investigates how *nonlinguistic* representations of actions can influence how children form initial *linguistic* representations of those actions when they are labeled with a novel verb.

More generally, most word learning studies to date have focused on isolated label-referent co-occurrences. But recent work by Smith and Yu and colleagues emphasizes that linguistic input is only a small part of input that children receive. In fact, a large amount of input simply involves visual experiences with referents, while label-referent co-occurrences are infrequent (Clerkin et al., 2017; Suanda et al., 2019; Yu et al., 2019). Our study is in line with the idea that researchers should not just focus their efforts on children’s labeled experiences, which are infrequent, but also on their unlabeled (visual) experiences, which are plentiful. By investigating prior experience in combination with iconic gesture, our study does not only aim to answer the question of how children learn words but also how children make use of different types of visual input they receive.

## Prior Unlabeled Experience Versus Delayed Labeling

Prior experience with unlabeled actions, which is the focus of the current study, differs from delayed labeling, in which children hear a label for an action immediately after the referent action has been demonstrated (e.g., Tomasello & Akhtar, 1995; Wakefield et al., 2018). The key feature of delayed labeling is that children do not see any other actions between seeing the referent action and hearing its label; thus, the referent action is the most plausible referent of the label. For example, previous research has shown that 2-year-old children can link a verb to its referent in delayed labeling situations in a verb learning task, in which a label is given immediately after the referent action is shown, and eye gaze cues direct children’s attention to the apparatus with which the action was performed (Tomasello & Akhtar, 1995). In the study by Tomasello and Akhtar (1995), a child and an experimenter took turns playing a merry-go-round game. In the following training phase, the experimenter modeled a novel target action with a novel object on the merry-go-round and readied the apparatus for the child’s turn. The experimenter then alternated her gaze between the child and the merry-go-round and provided a delayed language model (“It’s your turn now. *Widget*, Jason, *widget.*”). A control group followed the same procedure except that the child’s turn was preceded by neutral language (“Now it’s your turn, Jason, it’s your turn.”). Children in the control group heard the novel word (e.g., *widget*) for the first time in the comprehension test that followed the training phase. In the comprehension test, the experimenter set up the merry-go-round, three familiar objects, the novel object from the training phase, and a second novel object, and asked the children “Show me *widget*.”. Following this request, children in the experimental group generally performed the target action, whereas children in the control group indicated the objects. Thus, children could make use of delayed labeling (i.e., temporal adjacency cue) in combination with gaze alternation (i.e., nonverbal cue produced by an adult) in a verb learning task.

In contrast to delayed labeling, the key feature of prior experience with unlabeled actions is that children do see other actions between seeing the referent action and hearing its label. Thus, children cannot use temporal adjacency as a cue for linking a novel verb label to its referent action, and they will have to pick out the referent action out of many other actions from memory if they want to structurally align the referent action seen during labeling with the relevant action from prior experience.

## Possible Mechanisms

We distinguished three possible mechanisms for how prior experience with unlabeled actions and iconic gestures could promote children’s verb learning. First, children may structurally align (Gentner, 1982, 2003; Gentner & Markman, 1997; Markman & Gentner, 1993) an unlabeled exemplar and a labeled exemplar of the same action for verb learning. This *recall-event-and-align* mechanism suggests that when children encounter an action exemplar and a novel verb label (a) children recall the relevant exemplar (i.e., of the same action) from prior experience; (b) children structurally align (Gentner, 1982; 2003; Gentner & Markman, 1997; Markman & Gentner, 1993) the recalled unlabeled exemplar and the current labeled exemplar, which highlights the action as the shared component between exemplars; and (c) children interpret the highlighted action as the referent of the novel verb. This process requires children to be able to pick out relevant exemplars from memory.

Second, children may make use of prior experience with unlabeled actions *in combination* with iconic gestures that highlight the actions in the following way. This *gesture-for-action-memory* mechanism suggests that when children encounter an unlabeled action exemplar and an iconic gesture depicting the action in this exemplar, children’s attention is guided to the action by the information that is schematically depicted in gesture. This helps children to focus on action as a component of the exemplar and create a stable memory representation of the action. When children later go on to encounter a novel action exemplar (i.e., the same action performed in a different context) but now labeled with a novel verb, they can recognize the relevant action from prior experience in this exemplar. This makes the action stand out in the labeled exemplar, and as a result, children interpret the action as the referent of the novel verb.

Third, children may develop a general strategy for focusing on actions in the following way. This *gesture-for-general-strategy* mechanism suggests that when children encounter an action exemplar and an iconic gesture depicting the action in this exemplar, and this process is repeated for multiple different actions over time, iconic gesture may communicate to children the general strategy to pay attention to actions. Thus, when children encounter a labeled action exemplar, they may use this strategy to focus on actions more generally and as a result they may interpret actions as novel verb referents.

## The Current Study

We developed a novel verb learning task, in which children had the opportunity to link their prior experience with unlabeled actions from memory to labeled experiences with the same actions they encountered at a later point. Crucially, children did not encounter an unlabeled action and its label consecutively; children encountered multiple different unlabeled actions and performed a distraction task before any actions were labeled.

In Experiment 1, we manipulated prior experience with unlabeled actions and the gesture type that children saw with these unlabeled actions. The experiment had a *prior-experience*, *label*, and *test phase*. Gesture type was manipulated in the prior-experience phase, where all children were shown a block of six videos of unlabeled action exemplars. While viewing these exemplars, for half of the children, the experimenter produced iconic gestures that depicted the actions in the exemplars (iconic gesture conditions) and for half of the children the experimenter produced interactive gestures (Bavelas et al., 1992) that did not depict any aspect of the exemplars (interactive gesture conditions). Prior experience with unlabeled actions was manipulated in the label phase, where half of the children were taught novel verbs for actions they had seen in the prior-experience phase (relevant exemplar conditions), and half of the children were taught verbs for novel actions that they had not seen in the prior-experience phase (irrelevant exemplar conditions). In the *test phase*, children’s understanding of the novel verb meanings was tested in a two-alternative forced-choice task. Following the paradigm by Imai et al. (2008), children could correctly generalize each verb to a novel actor performing the action that was labeled in the label phase (same-action video) or incorrectly to the same actor as in the label phase performing a novel action (same-actor video).

## Predictions for Experiment 1

The *recall-event-and-align* mechanism predicts that children in the relevant exemplar conditions will successfully generalize more verbs than children in the irrelevant exemplar conditions, regardless of whether they see iconic or interactive gestures in the prior-experience phase, and that children in both relevant exemplar conditions will perform above chance.

The *gesture-for-action-memory* mechanism predicts that children in the relevant-iconic condition will successfully generalize more verbs than children in the other three conditions, and that children in the relevant-iconic condition will perform above chance.

The *gesture-for-general-strategy* mechanism predicts that children in the iconic gesture conditions will successfully generalize more verbs than children in the interactive gesture conditions, regardless of whether they see relevant or irrelevant exemplars in the prior-experience phase, and that children in both iconic gesture conditions will perform above chance.

## Experiment 1

### Method

#### Design

The experiment had a 2 × 2 between-participants design with prior experience (relevant exemplars vs. irrelevant exemplars) and gesture type (iconic gesture vs. interactive gesture) as independent variables. The dependent variable was children’s generalization performance for each of the six verbs in the two-alternative forced-choice test. We operationalized our dependent variable as a binary and coded children’s verb generalization performance as follows: when children pointed at the same-action video in the test phase, which was the correct extension of a given novel verb, they were given a score of 1, and when they pointed at the same-actor video in the test phase, which was the incorrect extension of a novel verb, they were given a score of 0.

#### Participants

The data were collected between March 23, 2016 and September 27, 2016. Our sample size was determined a priori using G*Power Version 3 (Faul et al., 2007) with an odds ratio of 2.30, α of .05, and power of .80. The final sample included 96 typically developing children (49 girls) between 36 and 48 months old (*M* = 41.14, *SD* = 3.71). There were 24 participants in each of the four groups. An additional nine children were tested but excluded from the analysis because they were too old on the day of testing (*N* = 6) or pointed exclusively to answers on the left or right side of the screen in the test phase (*N* = 3). Participants were recruited via 11 nurseries in the West-Midlands and Warwickshire areas (United Kingdom), and via a database of families who expressed their interest in taking part in language development research at the University of Warwick. Children in the relevant-iconic group were on average 41.22 months old (*SD* = 3.44), children in the relevant-interactive group 40.77 months old (*SD* = 3.72), children in the irrelevant-iconic group 41.21 months old (*SD* = 3.95), and children in the irrelevant-interactive group 41.37 months old (*SD* = 3.93). Children’s age in months did not differ significantly between the four groups, *F*(3, 92) = 0.11, *p* = .951. All children were exposed to the English language for at least 75% of the time (as indicated by their caregivers). The British Picture Vocabulary Scale–3 (BPVS3, Dunn et al., 2009) was used to assess children’s receptive vocabulary. This assessment was completed by 24 children in the relevant-iconic group, 22 children in the relevant-interactive group, 24 children in the irrelevant-iconic group, and 22 children in the irrelevant-interactive group, but four children were unable to finish this task. Children’s receptive vocabulary score did not differ significantly between the four groups, *F*(3, 88) = 1.30, *p* = .280, The relevant-iconic group had 11 boys and 13 girls and the other three groups each had 12 boys and 12 girls. Children’s gender did not differ significantly between the four groups, χ^2^(3) = 0.13, *p* = .989. Informed written consent was obtained from caregivers and participating nurseries for all participants. Nurseries received a voucher for their participation and children who participated in the research lab received a certificate and a toy. All children received a sticker bracelet as part of the task. All studies reported in this article received approval from the Humanities and Social Sciences Ethics Committee at the University of Warwick.

#### Materials

The stimulus videos, iconic gestures, interactive gestures, and novel words used in this study are described below.

##### Stimulus Videos

A set of 96 videos (4–15 s) depicting 24 actions was taken from the GRACE database (Aussems et al., 2017, 2018). Stimulus videos showed 24 actors (12 males, 12 females) moving across the length of a scene in an unusual manner using their feet, legs, or whole body. The actors always kept their arms by their side, their fingers pointing downward, parallel to their torso. The actions were normed based on the match between iconic gestures and actions, the similarity between different actors performing the same actions, the same actors performing different actions, and how unusual their movements were to adult native English speakers (for more detail, see Aussems et al., 2017, 2018). Ninety-six stimulus videos that received the best overall norming scores were selected, leading to a set of stimulus videos in which each of the 24 actions was depicted by two male actors and two female actors (see Table A1 in Appendix A for a list of the file names of the stimulus videos, which are available at http://wrap.warwick.ac.uk/78493/).

##### Iconic Gestures

**Figure 1 fig1:**
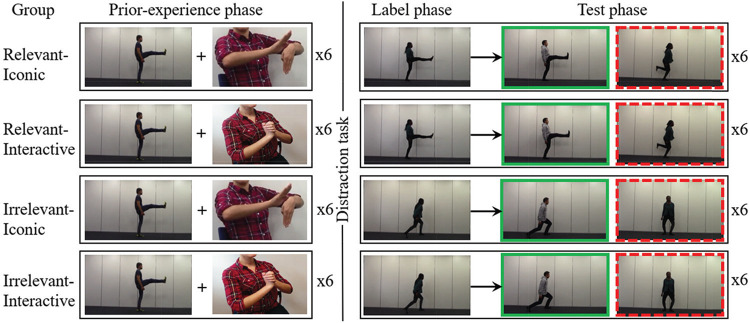
Four Between-Participant Conditions and Three Phases in the Procedure of Experiment 1 *Note*. From left to right, the overview shows each Group (Column 1) followed by examples of what children saw in the Prior-experience phase (Column 2), Label phase (Column 3), and Test phase (Column 4). The green solid line indicates the correct generalization and the red dotted line the incorrect generalization in the Test phase. For each group, six different actions were presented as a first block in the Prior-experience phase. In the second block, an action was labeled and immediately tested. This label-test procedure was repeated for six different actions. See the online article for the color version of this figure.

There were 24 iconic gestures (see Table A1 in Appendix A for a list of video examples of iconic gestures, which are available at http://wrap.warwick.ac.uk/78493/). Iconic gestures (McNeill, 1985, 1992) depicted how the actors in the stimulus videos moved. For example, if the actor in a stimulus video kicked their stretched legs up in an alternating fashion (Column 1 of Figure 1), then the experimenter produced an iconic gesture in which her right hand mapped onto the actor’s right leg and her left hand onto the actor’s left leg and she depicted the actor’s movement with her hands using the same shape (e.g., extended fingers represent stretched legs) and motion (e.g., upward flicking).[Fig fig1]

##### Interactive Gestures

There were three interactive gestures. Interactive gestures (Bavelas et al., 1992) were not related to any aspect of the action events in the stimulus videos but communicated excitement and surprise to engage the children in the interaction with the experimenter. Video examples of the three interactive gestures are available at https://osf.io/t52cn/.

##### Novel Words

The six novel words that were used to label the actions were *daxing, blicking, larping, stumming, pilking,* and *krading*. These made-up words follow the phonotactical rules of the English language and are commonly used in novel verb learning paradigms (e.g., Aussems & Kita, 2021; Childers, 2011; Maguire et al., 2008; Mumford & Kita, 2014; Naigles & Kako, 1993; Roseberry et al., 2009).

#### Procedure

The main experiment task consisted of three phases: a prior-experience, label, and test phase (see Figure 1). Most children also completed a receptive vocabulary task (BPVS3; Dunn et al., 2009) after the main experiment task. The procedures of the phases in the main experiment task and the vocabulary assessment are described below.

##### Prior-Experience Phase

In the prior-experience phase, children watched a first block of six videos of actions with the experimenter in a quiet area of the nursery or in the research lab. Each video was shown twice (on loop). When a video played the first time, the experimenter said “Wow! Look at what he (or she) is doing!,” and when the video played the second time the experimenter said “Oh! He (or she) is doing it again!” Note that the experimenter did not label the actions in the prior-experience phase. Depending on the condition, the experimenter accompanied these utterances with either an iconic (iconic gesture conditions) or interactive gesture (interactive gesture conditions). Figure 1 shows an example of an iconic gesture (Rows 1 and 3) and an interactive gesture (Rows 2 and 4). Each unusual movement was depicted with a unique iconic gesture and the experimenter rotated the three interactive gestures for the actions in the experiment task. The experimenter always sat on children’s left-hand side at a low table and produced iconic and interactive gestures live during the task. The experimenter looked at the stimulus videos on the screen while producing the gestures. All gestures were produced left to the center of the children’s field of vision, so that they could see the gestures and the stimulus videos on the computer screen simultaneously. Note that the experimenter *only* gestured in the prior-experience phase.

After the prior-experience phase, children spent 5 min decorating a paper wristband with colorful stickers. The experimenter asked the children to name the colors of the stickers and count the number of stickers on the wristband during this distraction task.

##### Label Phase and Test Phase

In the novel verb learning task, children were presented with a label phase and test phase in the following way. The experimenter presented the child with an action, labeled it, and immediately tested if the child could generalize the newly learned label to a novel situation. This label-test procedure was repeated for six different actions.

###### Label Phase

In the label phase, children watched videos of actions performed by novel actors (i.e., who had not appeared in the prior-experience phase). The experimenter now labeled the way these actors moved with a novel verb: “Look! He (or she) is [*daxing*]!” The video automatically played a second time, and the experimenter labeled the action again: “Wow! He (or she) is [*daxing*] again!” Depending on the condition, children were either taught labels for actions that they had seen during the prior-experience phase (relevant exemplar conditions) or novel actions that they had not seen before (irrelevant exemplar conditions).

###### Test Phase

Immediately after hearing the experimenter label an action with a novel verb, children’s knowledge of the verb’s meaning was tested in a test phase that showed two videos side-by-side (see Figure 1). One video showed a novel actor (never seen before in the experiment) performing the target action (same-action video), and the other video showed the actor from the label phase performing a novel action (same-actor video). The videos started playing automatically and the experimenter asked the child: “Which one is [*daxing*]?” The experimenter looked at the child while making this request and did not look at the screen until the child pointed at an answer, to ensure that eye gaze cues could not give away the correct answer. The two videos played continuously on loop until the child picked one. If the child did not respond or asked the experimenter whether a particular video showed [*daxing*], the question was repeated until one video was chosen. If the child pointed at both videos, the experimenter asked the child to pick one. Using on-screen buttons and Visual Basic for Applications (VBA), the data were automatically saved to an Excel spreadsheet.

##### Receptive Vocabulary Task

Finally, to obtain a measure of receptive vocabulary, children completed an adapted short version of the BPVS3 (Dunn et al., 2009) after the main experiment task. During this task, the experimenter showed four pictures on a quadrant and asked the child to point at the picture that showed the meaning of a word (e.g., “Where do you see [*word*]?”). If the child did not respond or asked the experimenter whether a particular picture showed the word’s referent, the question was repeated until one picture was chosen. If the child pointed at more than one picture, the experimenter asked the child to pick one. The task involved 17 nouns and 17 verbs (see Table B1 in Appendix B for a list of nouns and verbs). The experimenter recorded the children’s responses on paper.

#### Counterbalancing and Randomization

We created 24 versions of the experiment in which every stimulus video appeared equally in the prior-experience, label, and test phases (as target and distractor stimulus). In each experiment version, target videos appeared equally often on the left and right sides of the screen in the test phase and the target position was counterbalanced. We also counterbalanced the actors’ gender and male actors and female actors were equally represented in each experiment version. For a given action, if children saw a *male actor *in the prior-experience phase, then they saw a *female actor* in the label phase and test phase and a *second male actor* in the distractor video in the test phase (see Figure 1). If children saw a *female actor* in the prior-experience phase, then they saw a *male actor* in the label phase and test phase and a *second female actor* in the distractor video in the test phase. Out of the two male actors and two female actors who were performing the four actions, one actor was not shown (e.g., only three actors were needed to teach a verb, see Figure 1). In the same way, one of the four actions was not shown (e.g., maximally three actions were needed to teach a verb). This is because only two actions were shown per verb in the relevant exemplar conditions and three in the irrelevant exemplar conditions. We counterbalanced which actors and actions were shown. Because we had 24 actions and 24 actors to teach six verbs, a child never saw the same actor or action again for another verb. The 96 video clips used in this experiment are listed in Table A1 of Appendix A. The full counterbalancing and randomization spreadsheet of Experiment 1 is available via the Open Science Framework at https://osf.io/t52cn/.

The order in which the novel actions were shown in the prior-experience phase was prerandomized, and this order was the same in the prior-experience phase as in the label and test phases in the relevant exemplar conditions, and the same in the label and test phases in the irrelevant exemplar conditions.

Participants were pseudorandomly assigned to conditions based on their gender and age in months, before the experimenter met the children. We administered each experiment version to four children, one child in each condition, and this process was repeated for the next experiment version until all versions were completed by four children. Participants recruited from different nurseries and the research lab are therefore equally represented in each condition.

#### Data Analysis

Children’s verb generalization performance in each of the six test phases (binary, 1 = correct; i.e., choosing the same-action video; and 0 = incorrect; i.e., choosing the same-actor video) was entered into a mixed-effects logistic regression analysis using the *lme4* package (Bates et al., 2015) in the *R* software for statistical analyses (R Core Team, 2020). Fixed between-participants factors included prior experience (relevant exemplars vs. irrelevant exemplars) and gesture type (iconic gesture vs. interactive gesture). We specified a full model with a maximal random effects structure (cf. Barr et al., 2013); that is, a random intercept for participant, and a random slope and intercept, and the covariance between the two, for item (i.e., the stimulus videos that were labeled with a novel verb). To achieve model convergence, we dropped the random slopes over the main effects and interaction effect for item (cf. the procedure by Barr et al., 2013), and our final model included random intercepts only for participant and item. Likelihood ratio tests (χ^2^) were used to compare the full model with updated versions of the model that systematically excluded the main effects and interaction terms of interest. To explore the nature of the interaction effect, comparisons between two groups were made by running the model analysis over the subsets of the data that included the two groups of interest. Finally, the *SIGN.test()* function from the *BSDA* R package (Arnholt & Evans, 2017) was used to calculate one-sample sign tests for comparisons with chance and effect size *r* was calculated using Rosenthal’s formula (Rosenthal, 1994). The raw data and *R* Markdown files for all graphs and analyses are available via the Open Science Framework at https://osf.io/t52cn/.

### Results

**Figure 2 fig2:**
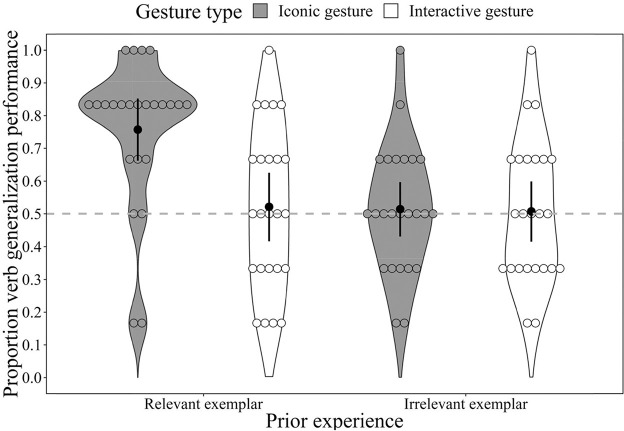
Main Findings of Experiment 1 *Note*. Proportion verb generalization performance (*y*-axis) organized by prior experience (*x*-axis) and gesture type (grey and white). Violins represent densities of the distribution in each group. Unfilled dots represent performances of individual children. Black dots represent group means. Error bars represent 95% confidence intervals around the group means. The dotted line at 0.5 indicates the chance level.

Figure 2 shows children’s verb generalization performance (in proportion) by prior-experience (relevant exemplars vs. irrelevant exemplars) and gesture type (iconic gesture vs. interactive). Children’s verb generalization performance for each of the six verbs (binary, 1 = correct; 0 = incorrect) was entered into a mixed-effects logistic regression analysis with prior experience and gesture type as fixed effects and participant and item as random effects (see Table 1 for the model output).[Fig fig2][Table tbl1]

The interaction effect between prior experience and gesture type on children’s verb generalization performance was significant, χ^2^(1) = 7.44, *p* = .006. To further analyze and interpret this interaction effect, we ran three separate mixed-effects logistic regression analyses over subsets of the data to compare the relevant-iconic group to the other three groups following our prediction. The relevant-iconic group successfully generalized more verbs than the relevant-interaction group, β = 1.21, *p* < .001, 95% CI [0.63, 1.89]; the irrelevant-iconic group, β = 1.12, *p* ≤ .001, 95% CI [0.57, 1.74]; and the irrelevant-interactive group, β = 1.20, *p* ≤ .001, 95% CI [0.61, 1.86] (see Figure 2).

Finally, one-sample sign tests revealed that the average performance .76 (95% CI [.68, .82]) of the relevant-iconic group was significantly above chance (test value = 0.50), *z* = −3.84, *p* < .001, *r* = .78. In contrast, the average performances were not significantly above chance in the relevant-interactive group: .52 (95% CI [.44, .60]) *z* = −0.00, *p* = .999, *r* = .00, the irrelevant-iconic group: .51 (95% CI [.43, .60]) *z* = −0.00, *p* = .999, *r* = 0.00, and the irrelevant-interactive group: .51 (95% CI [.42, .59]) *z* = −0.00, *p* = .999, *r* = 0.00.

### Discussion

Children in the relevant-iconic condition successfully generalized more verbs than children in the other three conditions. Moreover, only children in the relevant-iconic group performed above chance in the novel verb learning task, indicating that only children in this condition interpreted the novel verbs as action labels. This result is compatible with the *gesture-for-action-memory* mechanism, which predicted that that children would make use of prior experience with unlabeled actions in combination with iconic gestures that highlight the actions.

This result is not compatible with the *recall-event-and-align* mechanism. If children were able to structurally align (Gentner, 1982, 2003; Gentner & Markman, 1997; Markman & Gentner, 1993) an unlabeled and labeled exemplar of the same action (without help from iconic gesture), then children in both relevant exemplar conditions should have successfully generalized verbs more often than children in the irrelevant exemplar conditions, regardless of whether they saw iconic gestures or interactive gestures in the prior-experience phase, and children in both relevant exemplar conditions should have performed above chance. However, this was not the case.

This result is also not compatible with the *gesture-for-general-strategy* mechanism. If children developed a general strategy for focusing on actions, then children in both iconic gesture conditions should have successfully generalized verbs more often than children in the interactive gesture conditions, regardless of whether they saw relevant or irrelevant exemplars in the prior-experience phase, and children in both iconic gesture conditions should have performed above chance. However, this was not the case either.

Iconic gestures in the prior-experience phase of Experiment 1 depicted the actions. Thus, it is possible that children may have perceived iconic gestures as extra action exemplars. This *gesture-as-extra-exemplar* mechanism suggests that when children encounter an action exemplar and an iconic gesture depicting the action in the prior-experience phase, children could structurally align (Gentner, 1982, 2003; Gentner & Markman, 1997; Markman & Gentner, 1993) these two exemplars (i.e., the iconic gesture and a video) and pick up the invariance of action. Because the same structural alignment could be achieved when children encounter two relevant exemplars (i.e., two different videos) simultaneously, this would suggest that iconic gesture merely functions as an extra action exemplar. This alternative mechanism could explain the better performance in the relevant-iconic condition than in the relevant-interactive condition of Experiment 1; a difference we attributed to the *gesture-for-action-memory* mechanism.

Experiment 2 distinguished the *gesture-for-action-memory* mechanism and the *gesture-as-extra-exemplar* mechanism by comparing two types of prior experience with unlabeled actions: (a) one relevant exemplar with an iconic gesture that depicted the action (relevant-iconic condition); and (b) two relevant exemplars of the same action performed by different actors shown simultaneously and without gesture (two-relevant-exemplars condition).

### Predictions of Experiment 2

The *gesture-as-extra-exemplar* mechanism predicts that children will successfully generalize verbs equally well in the relevant-iconic condition and two-relevant-exemplars condition, and that both groups will perform above chance. However, we hypothesize instead that iconic gesture’s benefit goes beyond merely functioning as an extra relevant exemplar. Thus, we predict that, consistent with the *gesture-for-action-memory* mechanism, children in the relevant-iconic condition will successfully generalize more verbs than children in the two-relevant-exemplars condition, and that children in the relevant-iconic condition will perform above chance. This is because with two relevant exemplars, children must extract the invariant action component between those exemplars themselves via a bottom-up process. With one relevant exemplar and an iconic gesture, this task may be easier, because an iconic gesture (which is already schematized) can guide children’s attention to actions via a top-down process.

## Experiment 2

### Method

#### Design

The experiment had a between-participants design with type of prior experience as the independent variable and verb generalization performance as the dependent variable. Type of prior experience had two levels. One group of children was shown a relevant exemplar and an iconic gesture that depicted the action in this exemplar, in the same way as in the relevant-iconic condition of Experiment 1. The control group was shown two relevant exemplars of the same action (performed by different actors), and those exemplars were presented simultaneously without gesture (two-relevant-exemplars group). Verb generalization performance was again operationalized as a binary variable. We recorded whether children pointed at the same-action video (1 = correct) or the same-actor video (0 = incorrect) in the generalization task for each of the six verbs.

#### Participants

The data were collected between October 22, 2016 and February 14, 2017. We recruited the same number of children in each condition as in Experiment 1. The final sample included 48 typically developing children (22 girls, 26 boys) between 36 and 47 months old (*M* = 39.85, *SD* = 3.33). There were 24 children in each group. An additional four children were tested but were excluded from the analysis because they were too old on the day of testing (*N* = 1), pointed exclusively to answers on the left or right side of the screen in the test phase (*N* = 2), or were diagnosed with a language disorder (*N* = 1). Participants were recruited via the same nurseries as in Experiment 1, and via a database of families who expressed interest taking part in language development research at the University of Warwick, but none of the children had participated in Experiment 1. Children in the relevant-iconic group were on average 39.93 months old (*SD* = 3.85) and children in the two-relevant-exemplars group 39.76 months old (*SD* = 2.79). Children’s age in months did not differ significantly between the two groups, *t*(46) = 0.18, *p* = .861. All children were exposed to the English language for >75% of the time (as indicated by their caregivers). Twenty-two children in the relevant-iconic group and 22 children in the two-relevant-exemplars group completed the receptive vocabulary task (BPVS3; Dunn et al., 2009), but four children were unable to finish this task. Children’s receptive vocabulary score did not differ significantly between the two groups, *t*(42) = −0.34, *p* = .732. There were 12 boys and 12 girls in the relevant-iconic group and 14 boys and 10 girls in the two-relevant-exemplars group. Children’s gender did not differ significantly between the two groups, χ^2^(1) = 0.08, *p* = .772. Informed written consent was obtained from nurseries and caregivers for all child participants. Nurseries received a voucher for their participation and children who participated in the research lab received a certificate and a toy. All children received a sticker bracelet as part of the task.

#### Materials

The materials were the same as in Experiment 1.

#### Procedure

**Figure 3 fig3:**
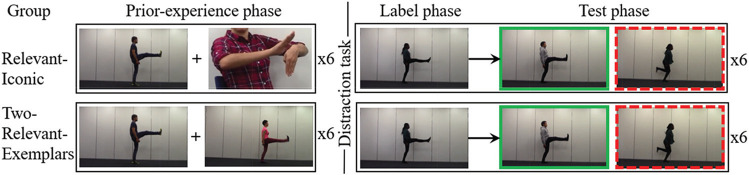
Two Between-Participant Conditions and Three Phases in the Procedure of Experiment 2 *Note*. The overview shows the Group (Column 1) followed by examples of what children saw in the Prior-experience phase (Column 2), Label phase (Column 3), and Test phase (Column 4). The green solid line indicates the correct generalization and the red dotted line the incorrect generalization in the Test phase. For each group, six different actions were presented as a block in the Prior-experience phase. In the second block, an action was labeled and immediately tested. This label-test procedure was repeated for six different actions. See the online article for the color version of this figure.

Children in the relevant-iconic group were tested using the same procedure as in Experiment 1. Children in the two-relevant-exemplars group followed the same procedure as the relevant-iconic group, except for the following differences in the prior-experience phase: children were shown two relevant exemplars (video clips of the same action performed by two different actors) simultaneously *without gesture* (see Figure 3). The videos were displayed side-by-side and when the videos played for the first time the experimenter said “Wow! Look at what *they* are doing!,” and when the videos played again the experimenter said “Oh! *They* are doing it again!” The label and test phases followed the same procedure as in the relevant-iconic group. The experimenter did not produce any gestures in the label and test phases for either group.[Fig fig3]

#### Counterbalancing and Randomization

Counterbalancing and randomization were the same as in Experiment 1, except for the following. To teach verbs with two relevant exemplars in the prior-experience phase, we needed to introduce a second exemplar that showed a different actor than in the rest of the experiment (for a given child). In Experiment 1, maximally three actors were needed to teach a verb out of the two male actors and two female actors selected for the two pairs of stimulus actions. As one actor was always unseen for any given verb in Experiment 1, we were able to introduce this actor in the second exemplar of the prior-experience phase of the two-relevant-exemplars condition. A full counterbalancing and randomization spreadsheet for Experiment 2 is available via the Open Science Framework at https://osf.io/t52cn/.

#### Data Analysis

The dependent variable verb generalization performance was analyzed in the same way as in Experiment 1. The fixed between-participants factor in our mixed-effects logistic regression analysis was type of prior experience (relevant-iconic vs. two-relevant-exemplars). We specified a full model with a maximal random effects structure (cf. Barr et al., 2013); that is, a random intercept for participant, and a random slope and intercept, and the covariance between the two, for item (i.e., the stimulus videos that were labeled with a novel verb). To achieve model convergence, we dropped the random slopes over the main effects and interaction effect for item (cf. the procedure by Barr et al., 2013). However, the model still did not converge. As the model output showed item variation did not explain any variance in the dependent variable, we dropped this random effect from the model altogether. The reported model therefore only included a random intercept for participant.

### Results

**Figure 4 fig4:**
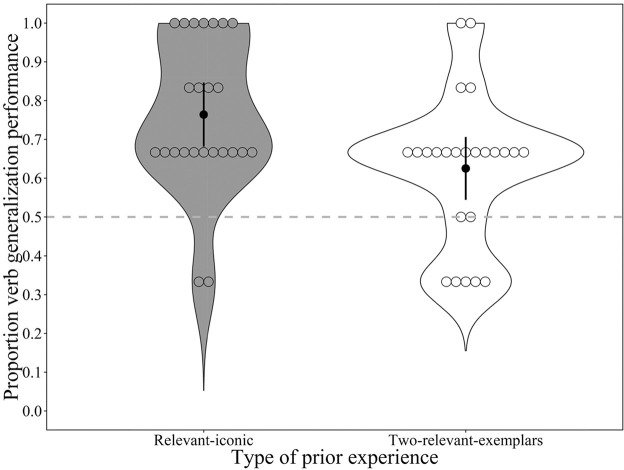
Main Findings of Experiment 2 *Note*. Verb generalization performance in proportion (*y*-axis) organized by type of prior experience (*x*-axis). Violins represent density. Unfilled dots represent performances of individual children. Black dots represent group means. Error bars represent 95% confidence intervals around the group means. Dotted line at 0.5 represents the chance level.

Figure 4 shows children’s verb generalization performance (in proportion) by type of prior experience with actions. Verb generalization performance for each of the six verbs (binary, 1 = correct; 0 = incorrect) was entered into a mixed-effects logistic regression analysis with type of prior experience as fixed effects and participant as random effect.[Fig fig4]

The main effect of type of prior experience on children’s verb generalization performance was significant, χ^2^(1) = 6.05, *p* = .014. Children in the relevant-iconic group successfully generalized more verbs than children in the two-relevant-exemplars group, β = 0.67, *p* = .013, 95% CI [0.14, 1.23].

Finally, one-sample sign tests revealed that the average performance .76, 95% CI [.69, .83] of children in the relevant-iconic group was significantly higher than chance (test value = 0.50), *z* = −4.13, *p* < .001, *r* = .84, and so was the average performance .63, 95% CI [.54, .70] of children in the two-relevant-exemplars group, *z* = −2.39, *p* = .017, *r* = .49.

### Discussion

Children in the relevant-iconic condition successfully generalized more verbs than children in the two-relevant-exemplars condition, and children in the relevant-iconic condition performed above chance. Importantly, this shows that the benefit of iconic gesture goes beyond merely functioning as extra relevant exemplars. This result is compatible with the *gesture-for-action-memory* mechanism, which predicted that that children would make use of prior experience with unlabeled actions in combination with iconic gestures that highlight the actions.

This result is not compatible with the *gesture-as-extra-exemplar* mechanism. If children simply perceived iconic gestures as extra exemplars, then children in both the relevant-iconic and two-relevant-exemplars conditions should have performed equally well, and both groups should have performed above chance. However, this was not the case.

But children in the two-relevant-exemplars condition performed above chance, indicating that they also interpreted the novel verbs as action labels. This shows that children were able to structurally align (Gentner, 1982, 2003; Gentner & Markman, 1997; Markman & Gentner, 1993) the two exemplars in the prior-experience phase, which helped them to extract the invariance of action at that point, and they used this prior experience for later verb learning.

## General Discussion

This study examined whether prior experience with unlabeled actions promotes 3-year-old children’s verb learning, and if so, what type of prior experience works best. In Experiment 1, we manipulated prior experience with unlabeled actions (relevant exemplars vs. irrelevant exemplars) and the gesture type (iconic gesture vs. interactive gesture) that children saw with this prior experience. Subsequently, we administered a novel verb learning task to the children to test how successfully they could generalize these newly learned verbs to novel events showing the referent actions. In Experiment 2, we further manipulated type of prior experience with unlabeled actions (relevant-iconic vs. two-relevant-exemplars) before administering the novel verb learning task. There are two key findings. First, children in the relevant-iconic condition successfully generalized more verbs than children in the other three conditions, and only children in the relevant-iconic condition performed above chance (Experiment 1). Thus, children were able to make use of prior experience with unlabeled actions in combination with iconic gestures that highlighted those actions. We argue that iconic gestures guided children’s attention to unlabeled actions during prior experience, and this helped children to create stable memory representations of those actions. Thus, this finding is compatible with the *gesture-for-action-memory* mechanism. Second, children in the relevant-iconic condition successfully generalized more verbs than children in the two-relevant-exemplars condition, although children in both conditions performed above chance (Experiment 2). Thus, the benefit of iconic gesture on children’s verb learning goes beyond merely functioning as an extra exemplar. We argue that this is because iconic gesture schematizes action (Aussems & Kita, 2019, 2021; de Ruiter, 2000; Goldin-Meadow, 2015; Kita, 2000; Kita et al., 2017; Novack et al., 2014; Novack & Goldin-Meadow, 2017), and it is a communicative signal, which prompts the recipient to search for a matching representation, triggering a top-down search for action. However, children in the two-relevant-exemplars condition also performed above chance. This suggests that these children structurally aligned (Gentner, 1982; 2003; Gentner & Markman, 1997; Markman & Gentner, 1993) the two unlabeled exemplars in the prior-experience phase and extracted the invariance of action, which also helped children to create stable action memory representations useful for the novel verb learning task; however, this process was not as effective for successful verb learning as seeing an iconic gesture with a relevant exemplar during prior experience. Thus, different cues for focusing children’s attention on actions in the prior-experience phase helped children to create stable memory representations of these actions, which became useful experience for later verb learning.

The results ruled out three other mechanisms for how prior experience with unlabeled actions promotes children’s verb learning. First, contrary to what the *recall-event-and-align* mechanism predicted, in Experiment 1, it was not the case that children in both relevant conditions successfully generalized more verbs than children in the irrelevant conditions, regardless of whether they saw iconic or interactive gestures in the prior-experience phase. Furthermore, only children in the relevant-iconic condition, but not the relevant-interactive condition, performed above chance. Thus, children were unable to structurally align (Gentner, 1982, 2003; Gentner & Markman, 1997; Markman & Gentner, 1993) an earlier single unlabeled exemplar and a later labeled exemplar of the same action for verb learning, without receiving a cue for focusing on action during prior experience. Second, contrary to what the *gesture-for-general-strategy* mechanism predicted, in Experiment 1, it was not the case that children in both iconic gesture conditions successfully generalized more verbs than children in the interactive gesture conditions, regardless of whether they saw relevant or irrelevant exemplars in the prior-experience phase. Furthermore, only children in the relevant-iconic condition, but not the irrelevant-iconic condition, performed above chance. Thus, children did not use iconic gesture to develop a general strategy for focusing on actions. Third, contrary to what the *gesture-as-extra-exemplar* mechanism predicted, in Experiment 2, it was not the case that children in the relevant-iconic and two-relevant-exemplars conditions successfully generalized verbs equally well. Thus, children did not simply perceive iconic gesture as an extra exemplar.

### How Did Children Learn Verbs in the Current Study?

We propose the following three steps for how children’s prior experience with unlabeled actions helped them to learn verbs. This model accounts for successful verb learning in the relevant-iconic conditions (Experiment 1 and 2) as well as the two-relevant-exemplars condition (Experiment 2).

Step 1: Children *focus* on the action component in a complex scene during the prior-experience phase. Seeing an iconic gesture depicting the action or seeing two exemplars of the same action simultaneously can facilitate this process. In this process, children form stable memory representations of actions, which are separate from the holistic representation of the whole event (i.e., the representations are independent from actors and other contextual information such as the scene’s background).

Step 2: Children *recognize* the actions seen in the labeled exemplars in the label-phase from their memory of unlabeled exemplars seen in the prior-experience phase. Because the actors always differed between exemplars of the same action, children cannot use the actor as a cue to recognize the same action. Thus, the children realize that one of the stable action representations encoded in memory in Step 1 matches the action in the event of the labeled exemplar based on the “sameness” of action.

Step 3: Children *extract* action from the labeled event because the recognition in Step 2 highlights the action. As a result, they interpret action as the referent of a novel verb.

Classic structural alignment (e.g., Gentner, 1982, 2003; Gentner & Markman, 1997; Markman & Gentner, 1993) is useful in Step 1. In both the relevant-iconic and two-relevant-exemplars conditions children compared and integrated information from two representations of the same action in the prior-experience phase, to focus on action and encode a stable memory representation. The relevant-iconic group did so with the help of an iconic gesture and a single exemplar, and the two-relevant-exemplars group did so based on the two exemplars.

### How Does This Study Advance Our Understanding of Children’s Word Learning?

The current study expands the word learning literature in two important ways. First, we investigated an important step of the verb learning process that has not been investigated before, namely, prior experience with unlabeled actions that later become verb referents. This is an important topic, because it is plausible that children possess and use such prior experience in naturalistic word learning situations. Our study shows that children can take advantage of prior experience with unlabeled exemplars in verb learning, but only when children were given a cue for focusing on action during prior experience. This cue could be a gestural cue triggering a top-down search for action via schematization (i.e., iconic gesture) or a structural cue triggering a bottom-up comparison of two events which may lead children to extract the invariance of action on their own (i.e., two relevant exemplars). Second, we demonstrated a new way in which children can take advantage of multiple exemplars for verb learning. The fact that the children in our study were able to use prior experience with *unlabeled* action exemplars for verb learning goes beyond findings from single exemplar word learning studies (Behrend, 1990; Forbes & Farrar, 1993, 1995; Goodrich & Hudson Kam, 2009; Imai et al., 2005, 2008; Kersten & Smith, 2002; Mumford & Kita, 2014; Naigles & Kako, 1993; Roseberry et al., 2009) and multiple exemplar word learning studies (Childers, 2011; Haryu et al., 2011; Maguire et al., 2008; Snape & Krott, 2018) in which each exemplar was always *labeled*. The idea that the status of children’s prior experience can change at the point of labeling is novel. The actions (without any associated labels) that children have seen before suddenly become relevant exemplars that children can take advantage of in verb learning. This is an additional ability that children may bring to word learning, beyond fast mapping (Carey & Bartlett, 1978) and cross-situational learning (Yu & Smith, 2008), both of which concern children’s exposure to word-referent combinations. Recent research has shown that nonlinguistic (visual) experience with referents is associated with children’s word learning (Clerkin et al., 2017; Suanda et al., 2019; Yu et al., 2019). More research is needed to investigate to what extent children use prior experience with unlabeled referents for later word learning in naturalistic settings, especially in combination with cues that highlight these referents, such as iconic gesture.

### A New Role for Gesture in Promoting Children’s Word Learning

The current findings show that gesture can promote word learning in a broader context than has previously been shown in the literature. Based on previous research we know that gestures can focus children’s attention on components of a complex event that are important for verb meaning (Mumford & Kita, 2014), and that this facilitates children’s word learning. However, the current study demonstrates a beneficial effect of iconic gesture that goes beyond indicating a referent of a concurrently presented novel word. After all, the gestures in our novel verb learning task were only presented with neutral language during prior experience and not during labeling. Thus, the benefit of gesture in our study goes beyond previous studies in which gesture was introduced at the point of labeling (Aussems & Kita, 2021; Goodrich & Hudson Kam, 2009; McGregor et al., 2009; Mumford & Kita, 2014). Future studies could investigate more ways of how iconic gesture can promote children’s word learning, for example, in combination with multiple labeled exemplars.

### Broader Implications for Learning From Gesture

It is possible that the mechanism through which gesture facilitates word learning can also facilitate learning in other domains. Gestures can show children how events are structured, highlighting relational concepts such as action. This mechanism can also be useful in the mathematical domain, for example, to facilitate children’s understanding of the perceptual structure of an equation (e.g., 8 + 6 + 2 = ___ + 2). When a teacher’s gestures indicate the key parts of the equation, such gestures can highlight the fact that the equation consists of two sides, left and right to the equal sign (Cook et al., 2013). This, in turn, helps children to understand that the two sides of an equation are related and must be made equal. Gesture thus has the capacity to highlight perceptual structure, and this beneficial effect extends beyond the word learning domain.

In Cook et al.’s (2013) mathematics learning study and Mumford and Kita’s (2014) verb learning study, gestures facilitated learning through linking information in multimodal learning situations, between speech, gesture, and the physical environment (Alibali et al., 2014). For example, in the study by Cook et al. (2013), the strategy to solve mathematical equations was provided in speech, as the experimenter said: “I want to make one side equal to the other side. Eight plus six plus two is sixteen, and fourteen plus two is sixteen. So, one side is equal to the other side.” Children who received these spoken instructions with gestures indicating the sides of the equation (one hand referred to one side of the equal sign and the other hand referred to the other side of the equal sign) solved more mathematical problems than children who had received the spoken instructions without gestures. In the study by Mumford and Kita (2014), the utterance produced by the experimenter described an event with a novel verb (*“Look! She’s (novel verb)-ing it!”*). Children who heard this utterance with an iconic gesture that depicted the verb referent in an exemplar video (e.g., manner of action), successfully interpreted the meaning of the novel verb. In both studies, only when a gesture linked the spoken language to the referent in the physical world (be it the sides of an equation or the manner of action in an exemplar video), this facilitated children’s learning.

Gesture’s function of grounding spoken language in the physical world is something that may occur across different learning contexts and domains. Iconic gestures could be considered as the “glue” that connects abstract ideas in speech to concrete objects in the physical world (Roth & Welzel, 2001). This makes abstract ideas expressed in spoken language more generalizable to child learners (Aussems & Kita, 2021; Novack et al., 2014). Iconic gestures could be considered as scaffolds for children to understand the link between abstract concepts and their concrete instantiations. That is, gesture may help children understand abstract ideas in such a way that they can apply these ideas in different contexts. This is because in the process of building a gesture-world link, children may come to understand how abstract ideas can be instantiated in the concrete physical world.

### Why Did Children in the Relevant-Interactive Condition Fail to Learn Verbs?

There are several possible reasons for why children did not successfully learn verbs in the relevant-interactive condition of Experiment 1. We highlight three of those reasons here. First and foremost, children may have formed unstable memory representations of actions. Without a cue for focusing on action in the prior-experience phase, the children may have encoded the action events in a holistic manner. That is, while encoding a rich action event as a whole, they may have encoded irrelevant details such as the actor, the background, and other aspects of the scene, in addition to the relevant information (i.e., action), which may have left children with unstable action memory representations that they could not use for later verb learning. Second, children may have been unable to structurally align the relevant unlabeled exemplar from memory and the labeled exemplar because there was no “tag” that indicated which unlabeled exemplar from memory was relevant to the labeled exemplar. Children had to pick out the relevant (i.e., same-action) unlabeled exemplar out of multiple exemplars stored in memory in the prior-experience phase. If the exemplars in the prior-experience phase had been labeled with the same novel verb, then this label could have served as a tag to link the two exemplars; however, all the exemplars in the prior-experience phase were unlabeled. Third, the saliency of seeing novel actors in the label-phase (compared with the actors seen in the prior-experience phase) may have prevented children from focusing on actions and mapping novel verbs to actions alone (i.e., without the actor in their semantic representation of a verb). After all, each exemplar of the same action always showed a novel actor in our paradigm. This may have led children to form semantic verb representations that included both actors and actions (cf. Imai et al., 2005), which could equally well be extended to same-action videos and same-actor videos in the test phase, resulting in chance performance.

### Why Did One Type of Prior Experience Promote Better Verb Learning Than the Other?

There may be two reasons for why children in Experiment 2, who saw a single relevant exemplar with an iconic gesture during the prior-experience phase, learned more verbs successfully than children who saw two relevant exemplars. First, iconic gestures represent actions in a schematic manner (Aussems & Kita, 2019, 2021; de Ruiter, 2000; Goldin-Meadow, 2015; Kita, 2000; Kita et al., 2017; Novack et al., 2014; Novack & Goldin-Meadow, 2017). That is, they focus on the key part of the movement and are stripped from unnecessary parts of the scene such as the characteristics of the actor (e.g., clothes, hair) and other irrelevant contextual information (e.g., the background of the scene). This may provide children with a “ready-to-use” schematized representation of action, which is useful for establishing a stable memory representation of action. In contrast, in the case of presenting two relevant exemplars simultaneously and without gesture, a schematic representation of action needs to be derived via structural alignment (e.g., Gentner, 1982, 2003; Gentner & Markman, 1997; Markman & Gentner, 1993) of the two exemplars. Second, an iconic gesture is a communicative signal. Thus, it prompts children to look for the referent of the gesture, which leads them to search for action in the accompanying exemplar video. In contrast, the simultaneous presentation of two exemplars merely invites children to compare the two exemplars but does not explicitly instruct or prompt children to do so. To summarize, iconic gesture triggers a top-down process for creating an action representation, whereas presenting two exemplars simultaneously triggers a less effective bottom-up process.

### Limitations

This study has two limitations. First, one could argue that interactive gestures focused children’s attention on the experimenter in Experiment 1 rather than on the stimulus videos, and this may have led to a difference between the iconic and interactive gesture conditions. As this study did not involve any eye tracking measures or video recordings of where children looked during the task, it is impossible to know for certain how the children divided their attention during the prior-experience phase of the task, and if this differed between the gesture conditions. However, we do know from previous research that seeing interactive gestures is not detrimental to children’s encoding of exemplar videos. In a study on event memory by Aussems and Kita (2019), 3-year-old children were shown the prior-experience phase of the current experiment task, using the exact same procedure and stimuli. One group of children saw the experimenter produce iconic gestures, another group saw interactive gestures, and a control group saw no gestures. After a short distraction task, children’s memory of the actors and the actions seen in the videos was tested in a two-alternative forced-choice task. Although the iconic gesture group remembered both actors and actions better than the interactive gesture group and the no gesture group, there was no statistically significant difference between the interactive gesture group and the no gesture group. Thus, interactive gestures did not negatively impact the way children encoded the exemplar videos, because when the experimenter did not produce any gestures, and therefore did not risk drawing children’s attention to herself and away from the stimuli, children’s event memory was the same as when she produced interactive gestures.

The second limitation is that exemplar variability could have led to a difference between the relevant-iconic and two-relevant-exemplars conditions in Experiment 2. Our relevant-iconic condition could be conceptualized as an extreme example of variability between two exemplars (if one considers both the iconic gesture and the video to be exemplars), whereas the two exemplars (i.e., videos) shown simultaneously in the two-exemplars condition were almost identical except for the actors. However, whether exemplar variability does or does not facilitate word learning is still a much-debated question, and the evidence is not clear-cut. For example, Maguire et al. (2008) showed that children generalized verbs more successfully when they saw the same actor perform the same action four times than children who saw four different actors perform the same action. In contrast, Perry et al. (2010) showed that it was in fact exemplar variability that supported verb generalization. Our prediction would be that learning verbs with an iconic gesture and a single exemplar video would lead to better generalization performance than two more varied exemplar videos. This is because iconic gesture depicts a “ready-to-use” schematic representation of the referent action, which can be applied to any exemplar of the same action, whereas children probably need to observe many more nonschematized exemplars to form such a generalizable representation themselves. In addition, iconic gesture is a communicative signal which guides children’s attention to the depicted actions a top-down manner, which makes forming a generalizable action representation easier than the bottom-up cue that two simultaneous exemplars provide, regardless of their variability.

## Conclusion

Our study shows that prior experience with unlabeled actions promotes 3-year-old children’s verb learning, provided that children get a cue for focusing on the relevant information (i.e., actions) during prior experience so that they can create stable memory representations of the actions that later become verb referents. Importantly, both iconic gestures and presenting two relevant exemplars simultaneously helped children to focus on actions during prior experience and promoted verb learning, although iconic gestures led to more successful verb learning than two simultaneous exemplars. Thus, when given cues for focusing on actions, children can form stable memory representations of those actions before any verb labels are introduced, which facilitates mapping those labels to their referents in future verb learning situations. To conclude, prior experience with unlabeled actions is an important first step in children’s verb learning process and the top-down cue that adults provide by producing iconic gestures to highlight actions as well as the bottom-up cue provided by presenting two simultaneous exemplars play a crucial role in this.

## Figures and Tables

**Table A1 tbl2:** List of Video 96 Files Taken From the GRACE Video Database (Aussems et al., 2017, 2018)

No.	Action exemplar	Iconic gesture	No.	Action exemplar	Iconic gesture
1.	01F_groining	00F_groining	49.	07M_creeping	00F_creeping
2.	01F_hopscotch	00F_hopscotch	50.	07M_crisscross	00F_crisscross
3.	01F_scurrying	00F_scurrying	51.	07M_marching	00F_marching
4.	01F_turning	00F_turning	52.	07M_wobbling	00F_wobbling
5.	01M_dragging	00F_dragging	53.	08F_dropping	00F_dropping
6.	01M_flicking	00F_flicking	54.	08F_folding	00F_folding
7.	01M_stomping	00F_stomping	55.	08F_grapevine	00F_grapevine
8.	01M_twisting	00F_twisting	56.	08F_shuffling	00F_shuffling
9.	02M_dropping	00F_dropping	57.	08M_hopping	00F_hopping
10.	02M_folding	00F_folding	58.	08M_skipping	00F_skipping
11.	02M_grapevining	00F_grapevining	59.	08M_swinging	00F_swinging
12.	02M_shuffling	00F_shuffling	60.	08M_trotting	00F_trotting
13.	03F_creeping	00F_creeping	61.	09F_bowing	00F_bowing
14.	03F_crisscross	00F_crisscross	62.	09F_mermaid	00F_mermaid
15.	03F_marching	00F_marching	63.	09F_overstep	00F_overstep
16.	03F_wobbling	00F_wobbling	64.	09F_skating	00F_skating
17.	03M_bowing	00F_bowing	65.	09M_creeping	00F_creeping
18.	03M_mermaid	00F_mermaid	66.	09M_crisscross	00F_crisscross
19.	03M_overstep	00F_overstep	67.	09M_marching	00F_marching
20.	03M_skating	00F_skating	68.	09M_wobbling	00F_wobbling
21.	04F_dragging	00F_dragging	69.	10F_groining	00F_groining
22.	04F_flicking	00F_flicking	70.	10F_hopscotching	00F_hopscotching
23.	04F_stomping	00F_stomping	71.	10F_scurrying	00F_scurrying
24.	04F_twisting	00F_twisting	72.	10F_turning	00F_turning
25.	04M_groining	00F_groining	73.	10M_dragging	00F_dragging
26.	04M_hopscotch	00F_hopscotch	74.	10M_flicking	00F_flicking
27.	04M_scurrying	00F_scurrying	75.	10M_stomping	00F_stomping
28.	04M_turning	00F_turning	76.	10M_twisting	00F_twisting
29.	05F_hopping	00F_hopping	77.	11F_dragging	00F_dragging
30.	05F_skipping	00F_skipping	78.	11F_flicking	00F_flicking
31.	05F_swinging	00F_swinging	79.	11F_stomping	00F_stomping
32.	05F_trotting	00F_trotting	80.	11F_twisting	00F_twisting
33.	05M_hopping	00F_hopping	81.	11M_groining	00F_groining
34.	05M_skipping	00F_skipping	82.	11M_hopscotch	00F_hopscotch
35.	05M_swinging	00F_swinging	83.	11M_scurrying	00F_scurrying
36.	05M_trotting	00F_trotting	84.	11M_turning	00F_turning
37.	06F_creeping	00F_creeping	85.	12F_bowing	00F_bowing
38.	06F_crisscross	00F_crisscross	86.	12F_mermaiding	00F_mermaiding
39.	06F_marching	00F_crisscrossing	87.	12F_overstep	00F_overstep
40.	06F_wobbling	00F_marching	88.	12F_skating	00F_skating
41.	06M_bowing	00F_wobbling	89.	12M_dropping	00F_dropping
42.	06M_mermaid	00F_bowing	90.	12M_folding	00F_folding
43.	06M_overstep	00F_mermaid	91.	12M_grapevine	00F_grapevine
44.	06M_skating	00F_overstep	92.	12M_shuffling	00F_shuffling
45.	07F_dropping	00F_skating	93.	13F_hopping	00F_hopping
46.	07F_folding	00F_dropping	94.	13F_skipping	00F_skipping
47.	07F_grapevine	00F_folding	95.	13F_swinging	00F_swinging
48.	07F_shuffling	00F_grapevine	96.	13F_trotting	00F_trotting
*Note*. The Action exemplar columns lists the names of the video files of the action events as they were shown in the task. The Iconic gesture columns list the names of the video files of examples of iconic gestures, which were produced live by the experimenter during the experiment. Videos are available in MP4 Format via http://wrap.warwick.ac.uk/78493/. The file names in this table contain a reference to an actor (No. 00-13), their gender (F = female, M = male), and a short-hand label for manner of locomotion (e.g., creeping).					

**Table B1 tbl3:** Nouns and Verbs Taken From the British Picture Vocabulary III (BPVS-3, Dunn et al., 2009)

No.	Nouns BPVS3 measure	% Correct *M* (*SD*) Exp. 1 & 2 (*N* = 136)	No.	Verbs BPVS3 measure	% Correct *M* (*SD*) Exp. 1 & 2 (*N* = 136)
1.	Spoon	99.26 (0.73)	1.	Jumping	99.26 (0.73)
2.	Cat	99.26 (0.07)	2.	Drinking	99.79 (0.13)
3.	Money	96.32 (0.16)	3.	Swimming	91.91 (0.23)
4.	Panda	83.01 (0.38)	4.	Dancing	83.82 (0.32)
5.	Nest	79.41 (3.47)	5.	Dressing	75.00 (3.71)
6.	Mountain	88.97 (2.69)	6.	Hopping	81.62 (3.32)
7.	Rectangle	63.24 (4.13)	7.	Juggling	75.74 (3.68)
8.	Elbow	63.97 (4.12)	8.	Sawing	71.32 (3.88)
9.	Diamond	71.32 (3.88)	9.	Sharing	53.68 (4.28)
10.	Target	52.94 (4.28)	10.	Diving	65.44 (4.08)
11.	Desk	41.18 (4.22)	11.	Delivering	72.79 (3.82)
12.	Astronaut	80.88 (3.37)	12.	Jogging	55.15 (4.26)
13.	Chimney	61.76 (4.17)	13.	Measuring	52.59 (4.28)
14.	Package	55.88 (4.26)	14.	Tearing	43.38 (4.25)
15.	Harp	41.91 (4.23)	15.	Floating	53.68 (4.28)
16.	Vehicle	33.82 (4.06)	16.	Dripping	47.79 (4.28)
17.	Brain	22.06 (3.56)	17.	Sorting	34.56 (4.08)
*Note*. The column Nouns lists the 17 nouns and the column Verbs the 17 verbs taken from the British Picture Vocabulary III (BPVS-3, Dunn et al., 2009). The column % Correct *M* (*SD*) lists the means and standard deviations of the percentage of children (across the two experiments) who pointed at the correct referent for a given noun or verb. *N* = the number of children.					

**Table 1 tbl1:** Model Output of the Mixed-Effects Logistic Regression Analysis of Experiment 1

Fixed effects	β	*SE*	*z*	*LL*	*UL*
(Intercept)	1.22***	0.23	5.33	0.79	1.69
Prior experience (Relevant vs. irrelevant)	−1.16***	0.29	−3.94	−1.76	−0.59
Gesture type (Iconic vs. interactive)	−1.13***	0.29	−3.84	−1.73	−0.56
Prior Experience × Gesture Type (Relevant-iconic vs. the other three conditions)	1.10**	0.40	2.74	0.31	1.91
*Note*. β = β estimates; *SE* = standard error around the β estimates; *z* = *z-*test value; *LL* = lower limit and *UL* = upper limit of the 95% confidence interval around the β estimates. Model specification: glmer(verb generalization performance ∼ Prior Experience × Gesture Type + (1 | participant) + (1 | item). Dummy coding was used for the fixed effects, and the reference level is indicated in parentheses for each effect.					
** *p* < .01. *** *p* < .001.					

## A Stimuli Used in Experiments 1 and 2

[Table tbl2]

## B Receptive Vocabulary Task

[Table tbl3]
