# Correction to Aussems, Mumford, and Kita (2021)

**DOI:** 10.1037/xge0001163

**Published:** 2022-01-13

In the article “Prior Experience With Unlabeled Actions Promotes 3-Year-Old Children’s Verb Learning” by Suzanne Aussems, Katherine H. Mumford, and Sotaro Kita (*Journal of Experimental Psychology: General*. Advance online publication. July 15, 2021. http://dx.doi.org/10.1037/xge0001071), acknowledgment of and formatting for Economic and Social Research Council funding was omitted. The author note and copyright line now reflect the standard acknowledgment of and formatting for the funding received for this article.

All versions of this article have been corrected.

